# Calpain-2 protein influences *chikungunya virus* replication and regulates vimentin rearrangement caused by *chikungunya virus* infection

**DOI:** 10.3389/fmicb.2023.1229576

**Published:** 2023-10-19

**Authors:** Jia Li, Kang Zheng, Huilong Shen, Hua Wu, Chengsong Wan, Renli Zhang, Zhimin Liu

**Affiliations:** ^1^Department of Clinical Laboratory, Affiliated Hengyang Hospital of Southern Medical University, Hengyang Central Hospital, Hengyang, China; ^2^Institute of Pathogenic Organisms, Shenzhen Center for Disease Control and Prevention, Shenzhen, China; ^3^Biosafety Level 3 Laboratory, School of Public Health, Southern Medical University, Guangzhou, China

**Keywords:** chikungunya virus, calpain-2 protein, vimentin protein, replication, antiviral strategies

## Abstract

Chikungunya fever (CHIF), a vector-borne disease transmitted mainly by *Aedes albopictus* and *Aedes aegypti*, is caused by *Chikungunya virus* (CHIKV) infection. To date, it is estimated that 39% of the world’s population is at risk of infection for living in countries and regions where CHIKV is endemic. However, at present, the cellular receptors of CHIKV remains not clear, and there are no specific drugs and vaccines for CHIF. Here, the cytotoxicity of calpain-2 protein activity inhibitor III and specific siRNA was detected by MTT assays. The replication of CHIKV was detected by qPCR amplification and plaque assay. Western blot was used to determine the level of the calpain-2 protein and vimentin protein. Immunofluorescence was also operated for detecting the rearrangement of vimentin protein. Our results indicated that calpain-2 protein activity inhibitor III and specific siRNA might suppress CHIKV replication. Furthermore, CHIKV infection led to vimentin remodeling and formation of cage-like structures, which could be inhibited by the inhibitor III. In summary, we confirmed that calpain-2 protein influenced chikungunya virus replication and regulated vimentin rearrangement caused by chikungunya virus infection, which could be important for understanding the biological significance of CHIKV replication and the future development of antiviral strategies.

## Introduction

Chikungunya fever (CHIF), a vector-borne disease transmitted by *Aedes albopictus* and *Aedes aegypti*, is caused by *Chikungunya virus* (CHIKV) (Vairo et al., 2019). CHIKV was first identified in 1952–1953 during an outbreak in the Makund Plateau in southern region of Tanzania, as a member of the genus alphavirus of the family togaviridae (Robinson, 1955; Burt et al., 2017). CHIKV is a non-segmented single-stranded positive-strand RNA virus. Its genome length is about 12 kb, encoding 5 structural proteins (C, E1, E2, E3, 6 K) and 4 non-structural proteins (nsP1, nsP2, nsP3, nsP4) (Burt et al., 2012; Schnierle, 2019). Before CHIKV enters the cell through clathrin-mediated endocytosis, it bound to an unknown receptor on the host cell membrane. After the fusion of the virus and the host cell membrane, the nucleocapsid is released into the cytoplasm. The corresponding protein is translated, following by the assembly of the components into mature virus particles, which are released to the outside of the cell in a budding manner (Leung et al., 2011; Rashad et al., 2014; van Duijl-Richter et al., 2015; Bartholomeeusen et al., 2018; Schnierle, 2019). The main symptoms during an acute attack of chikungunya are sudden high fever and severe joint pain and myalgia that may last for months or years, accompanied by headache, photophobia and rash, which can also cause viremia in severe cases (Borgherini et al., 2007; Rezza et al., 2007; Lemant et al., 2008; Queyriaux et al., 2008). CHIKV has experienced outbreaks in many places, such as Thailand in 1960, Sint Maarten in 2013, Bangladesh in 2017, etc. It is spreading at an alarming rate across the world and has affected millions of people in tropical and subtropical regions, which has brought great harm and challenges to global health and public health (Nimmannitya et al., 1969; Cassadou et al., 2014; Hossain et al., 2018). Up to now, there is no effective drug-specific vaccine against chikungunya, which makes it urgent to understand the pathogenic mechanism of chikungunya virus.

The calpain family is a class of Ca^2+^-dependent, non-lysosomal neutral cysteine proteases that are localized in the cytoplasm and usually exist as inactive zymogens (Xu et al., 2019). Among them, the two prototype calpains are calpain-1 and calpain-2 protein (also known as μ-calpain and m-calpain, respectively). They are generally expressed in mammalian cells (Goll et al., 2003; Zatz and Starling, 2005; Sorimachi et al., 2011). Both calpain-1 and calpain-2 protein are heterodimers, consisting of 28 KD regulatory subunit and 80 KD catalytic subunit (Arthur et al., 2000; Campbell and Davies, 2012). Their target proteins are almost the same and the major difference is their different requirements for Ca^2+^ concentration during activation: 3–50 μM for calpain-1 protein and 400–800 μM for calpain-2 protein (GOLL et al., 2003; Upla et al., 2008). Calpains have a large number of target proteins, many of which are actin-related proteins and cytoskeleton proteins, such as α-cytosin, ankle protein, paxillin, etc. In addition, calpains can also degrade the cytoplasmic structure of integrin, which reflects that calpains may be involved in regulating cytoskeleton-membrane interactions, intracellular vesicle transport, and cytoskeleton remodeling (Pfaff et al., 1999; Schneider et al., 2012; Karpe et al., 2016).

Studies have shown that calpain protein affects the replication process of a variety of viruses. After Echovirus type I (EV1) infected cells, the activity of calpain protein in the cytoplasm was enhanced, and the formation of the replication complex of EV1 was closely related to the activity of calpain protein (Upla et al., 2008). The calpain protein inhibitor MDL28170 could inhibit cathepsin L-mediated processing of the S protein of severe acute respiratory syndrome coronavirus (SARS-CoV), thereby preventing the process of virus entry into cells mediated by the S protein of SARS-CoV (Simmons et al., 2005; Schneider et al., 2012). In the acute myocarditis model caused by Coxsackievirus B virus type 3 (CVB3), the expression of calpain protein was increased, and in the host cardiomyocytes, calpain protein could promote the replication of CVB3 by regulating cell apoptosis (Li et al., 2014). In addition, CHIKV replication increased the activity of the proteasome in the cell, and virus replication depended on the activity of the proteasome and calpain-2 protein (Wang et al., 2015).

Vimentin is a type III intermediate filament cytoskeleton protein. Its monomer is about 54 kDa and consists of four α-helix fragments (Herrmann and Aebi, 2016; Ramos et al., 2020). Vimentin is widely present in cells and participates in the movement of cells and the maintenance of cell structure, as well as the infection process of a variety of viruses (Challa and Stefanovic, 2011; Fay and Panté, 2013; Haolong et al., 2013; Turkki et al., 2013). A study reported that vimentin played a crucial part in the process of SARS-CoV virus entry into cells by interacting with the S protein of angiotensin-converting enzyme (ACE) 2 (Yu et al., 2016). In addition, the envelope protein of dengue virus in endothelial cells interacted with the vimentin rod domain to promote virus absorption and subsequent infection (Yang et al., 2016). For the alphavirus of the Togavirus family, studies showed that virus infection induced the destruction of the action network, but did not destroy the intermediate filaments of the vimentin, which declared that the vimentin may play an important role in the replication of alphaviruses (Laakkonen et al., 1998). At the same time, in the replication process of SINV, which was closely related to CHIKV, vimentin was related to its non-structural protein nsP3, and could promote the formation of RC of SINV (Frolova et al., 2006). In the process of CHIKV replication, specific siRNA silencing the expression of vimentin gene could lead to a significant decrease in infectious CHIKV titer. More interestingly, CHIKV replication could also cause the rearrangement of vimentin, so that vimentin could form an anchor around RC Network to ensure the effective replication of the virus itself (Issac et al., 2014).

In summary, calpain protein is involved in the replication or pathogenic process of a variety of viruses, and many of them are single-stranded positive-stranded RNA viruses that enter host cells through endocytosis like CHIKV. The role of calpain-2 protein in virus replication is particularly prominent, and can even be used as a potential therapeutic target for some viruses (Blanc et al., 2016; Karpe et al., 2016). But how does calpain-2 protein affect CHIKV replication? Is this process related to vimentin? This is an issue that has not yet been clarified. Therefore, this study analyzed the expression and activity of calpain-2 protein before and after CHIKV infection, and at the same time, explored whether calpain-2 protein would affect the replication of CHIKV and its specific mechanism by inhibiting the expression and activity of calpain-2 protein. It can lay a theoretical foundation for exploring the pathogenic mechanism of CHIKV and provide a scientific basis for the prevention, control and treatment of CHIF.

## Materials and methods

### Viruses and cell culture

*Chikungunya virus* (GenBank accession number: MG_664850.1) was stored in the laboratory of Shenzhen Center for Disease Control and Prevention as previously described (Zhang et al., 2018a,b). Hela cells (ATCC CCL-2) and Vero cells (ATCC CCL-81) were used in the experiments and cultured in Dulbecco’s modification of Eagle’s medium (DMEM) (Gibco) supplemented with 10% fetal bovine sera (FBS) (Gibco), 100 U/mL penicillin (Gibco), and 100 μg/mL streptomycin (Gibco) at 37°C with 5% CO_2_. All experiments with CHIKV live virus were done in a biosafety level 3 facility of Shenzhen Center for Disease Control and Prevention. The studies involving human participants were reviewed and approved by Ethics Committee of Southern Medical University (K2021016). The patients/participants provided their written informed consent to participate in this study.

### Reagents and antibodies

Calpain inhibitor III (MDL28170; CI III) were purchased from Calbiochem/Merck, dissolved in dimethylsulfoxide (DMSO) (Sigma-Aldrich) and stored at −20°C. The following antibodies against cellular structures were used: rabbit monoclonal anti-vimentin antibody (CST), anti-GAPDH antibody (Abcam); rat monoclonal anti-tubulin antibody (Abcam); mouse monoclonal anti-calpain 2 antibody (Abcam), anti-β-actin antibody (Abcam). Goat anti-rabbit, rabbit anti-rat and goat anti-mouse antibodies were from Abcam. Polyvinylidene difluoride (PVDF) membranes were obtained from Millipore. The chemiluminescent Western blotting detection reagent ECL Plus was purchased from Thermo Scientific™. The Calpain Activity Assay Kit (Fluorometric) was from Abcam.

### Infection with CHIKV

Twenty-four hours after seeding, cells were pretreated with different chemical compounds in FCS-free medium for 1 h. After pre-treatment, cells were washed with phosphate-buffered saline (PBS) (Double-helix Biotech), and CHIKV infection with or without compounds was performed in 1 mL FCS-free medium at 37°C under the multiplicity of infection (MOI) required for the experiment. After a certain incubation time, the inoculum was removed and the cells were washed 3 times with PBS, then 3 mL of complete growth medium was added to continue the incubation with or without compounds.

### Plaque assay

Vero cells in 12-well plates were infected with 5 serial dilutions of virus supernatants in FCS-free medium and incubated at 37°C for 1 h. After that, 3% (W/V) low-melting agarose (Sangon) and DMEM medium with 2% FBS were mixed at a ratio of 1:2 to make an agar cover and added to 12-well plates. The plates were cooled and solidified at room temperature, and was incubated at 37°C. After 2–3 days of incubation, cells were fixed with 4% formaldehyde (Sangon), stained with crystal violet solution (Sigma-Aldrich), and then the plaques were counted.

### siRNA-mediated protein depletion

Short interfering RNA (siRNA) duplexes for human calpain 2 proteins (CAPN-1189; GenBank accession number: NM_001748) were synthesized by Sangon. siRNA was GGAUGUCUUUCAGUGACUUTT (calpain 2–1) and AAGUCACUGAAAGACAUCCTT (calpain 2–2). A nontargeting scramble siRNA (NC) was used as a control. Hela cells were transfected with 20 pmol of siRNAs using Lipofectamine 2000 (Sigma-Aldrich) according to the manufacturer’s protocol. Briefly, 400 μL of OPTI-MEM (Gibco) was added to the cells in the plates. 1 μL of Lipofectamine 2000 (20 μM) was added to 50 μL of OPTI-MEM and incubated for 5 min at room temperature. Similarly, 1 μL of siRNA (20 μM) and 50 μL of OPTI-MEM were incubated for 5 min and mixed with diluted Lipofectamine 2000 for an additional 20 min at room temperature. The mixture was added to the cells and incubated at 37°C for 6 h. After that, the supernatant was changed to 0.5 mL of DMEM containing 5% FCS. Forty-eight hours after transfection, cells were lysed for immunoblot analysis or infected with CHIKV. After incubation for 16 h, viral titers were determined by plaque assay and the amount of RNA was detected by TaqMan qPCR method.

### Cell lysis and immunoblot analysis

Hela cells were washed with pre-chilled PBS and scraped into RIPA lysis buffer (Sangon). After being incubated on ice for 30 min, the cell lysate was centrifuged at 14,000 rpm for 10 min. The supernatant was aliquoted and stored at −80°C. For Western blot analysis, the protein was thawed at 4°C and the total protein concentration was determined using the Pierce™ Rapid Gold BCA Protein Quantification Kit (Thermo Scientific™). After being boiled at 100°C for 5 min, an equal amount of protein was analyzed by 10% SDS-PAGE (Sangon). The protein was electrotransferred onto a PVDF membrane, which was blocked with skim milk powder (Sangon) (5%, W/V) in Trisbuffered saline (TBST) (Sangon) (0.05% Tween 20, 100 mM NaCl, 10 mM Tris–HCl; pH 7.8). After the incubation with appropriate primary and secondary horseradish peroxidase (HRP)-conjugated antibodies, protein bands were detected using a chemiluminescence blot (ECL) kit (Thermo Scientific™).

### Cytotoxicity assay

Hela cells were seeded in 96-well plates (1.2 × 10^4^ cells/well) and transfected with 40 nM of siRNAs or treated with various concentrations of inhibitor III. A 100 μL volume of MTT (3-[4,5-dimethylthiazol-2-yl]-2,5-diphenyltetrazolium bromide) (500 μg/mL; Sigma-Aldrich) reagent was added to each well. After incubation at 37°C for 4 h, the cell supernatant of each well was removed. A 100 μL volume of DMSO was added to each well, followed by 10 min of shaking. Absorbance values were measured at a wavelength of 570 nm.

### Quantitative RT-PCR

RNA was extracted from Hela cells using AxyPrep Multisource Total RNA Miniprep Kit (Axygen) and reversed into cDNA with the ReverTra Ace qPCR RT Kit (TOYOBO). RNA prepared by the column method contains genomic DNA, so total RNA was treated with DNase I before transcription. PCR was performed with an ABI 7500 instrument (Thermo Scientific™) using a reaction mixture containing QuantiTect SYBR green PCR mix (Qiagen), 250 nM of each of the primers, and 1 μL cDNA product. Quantitative RT-PCR was performed using primers described as follows: CAPN2-F: TTTGTGCGGTGTTTGGTCC; CAPN2-R: AAGTTCAAGGTCCAGATACAAAGTG; β-actin-F: GGCGGCACCACCATGTACCCT; β-actin-R: AGGGGCCGGACTCGTCATACT; CHIKV-E1-F: AAGCTCCGCGTCCTTTACCAAG; CHIKV-E1-R: AATTTAGCGTCCTTAACTGTGACGG. Amplification was completed according to the following steps: 1 cycles of 95°C for 1 min; 40 cycles of 95°C for 15 s, 56°C for 15 s and 72°C for 45 s; 1 cycles of 95°C for 15 s, 60°C for 1 min, 95°C for 15 s, 60°C for 15 s. The specificity of the reaction was confirmed by melting curve analysis. The mRNA expression for target genes was normalized using the mean (for one housekeeping (HK) gene: β-actin. The ΔΔCt value was calculated as follows: ΔΔCt = 2^-[Mean Ct (target gene)-Mean Ct (β-actin)]^.

### Calpain activity assay

Hela cells (initial recommendation 1–2 × 10^6^ cells) were harvested for each assay and washed with cold PBS. Hela cells were resuspended in 100 μL of Extraction Buffer provided by the kit, and then the concentration of protein was measured. For each treatment group, 100ug of protein was taken and diluted with lysate in the kit to 85 μL. 10 μL 10 × reaction buffer from the kit was added and 5 μL fluorescent substrate of calpain provided by the kit was added subsequently under dark condition. After the reaction, the fluorescence light absorption values at 400 mn emission wavelength and 505 nm excitation wavelength were measured with a fluorescent enzyme spectrometer. The final results were represented with Relative Fluorescent Unit (RFU) and compared between groups.

### Indirect immunofluorescence assay (IFA)

Hela cells (7 × 10^4^ cells) were seeded on the coverslips and fixed with permeabilization solution. After fixation, the cells were then incubated with mouse anti-Chikungunya virus antibody (Abcam, 1:25 dilution) and rabbit anti-vimentin antibody (CST, 1:100 dilution), followed by incubation with Goat Anti-Mouse IgG H&L (Alexa Fluor® 488) (abcam, 1:200 dilution) and Donkey Anti-Rabbit IgG H&L (Alexa Fluor® 568) (abcam, 1:200 dilution). Cell nuclei were stained with 4′,6′-diamidino-2-phenylindole (DAPI) (Invitrogen). The samples were viewed using Laser confocal microscopy system (Leica, TCS SP5) under an oil mirror. The scale bars are 5 μm and resolution on imaging data is 512 × 512.

### Statistical analysis

Statistical analysis was performed with GraphPad Prism 7 software using the unpaired two-tailed *t* test for pairwise comparisons. Statistical significance was expressed as the following: *p < 0.05; **p < 0.01; ***p < 0.001; ****p < 0.0001; and ns, not significant.

## Results

### The activity inhibitor III of calpain-2 protein could effectively suppress the replication of CHIKV

**Figure 1:**
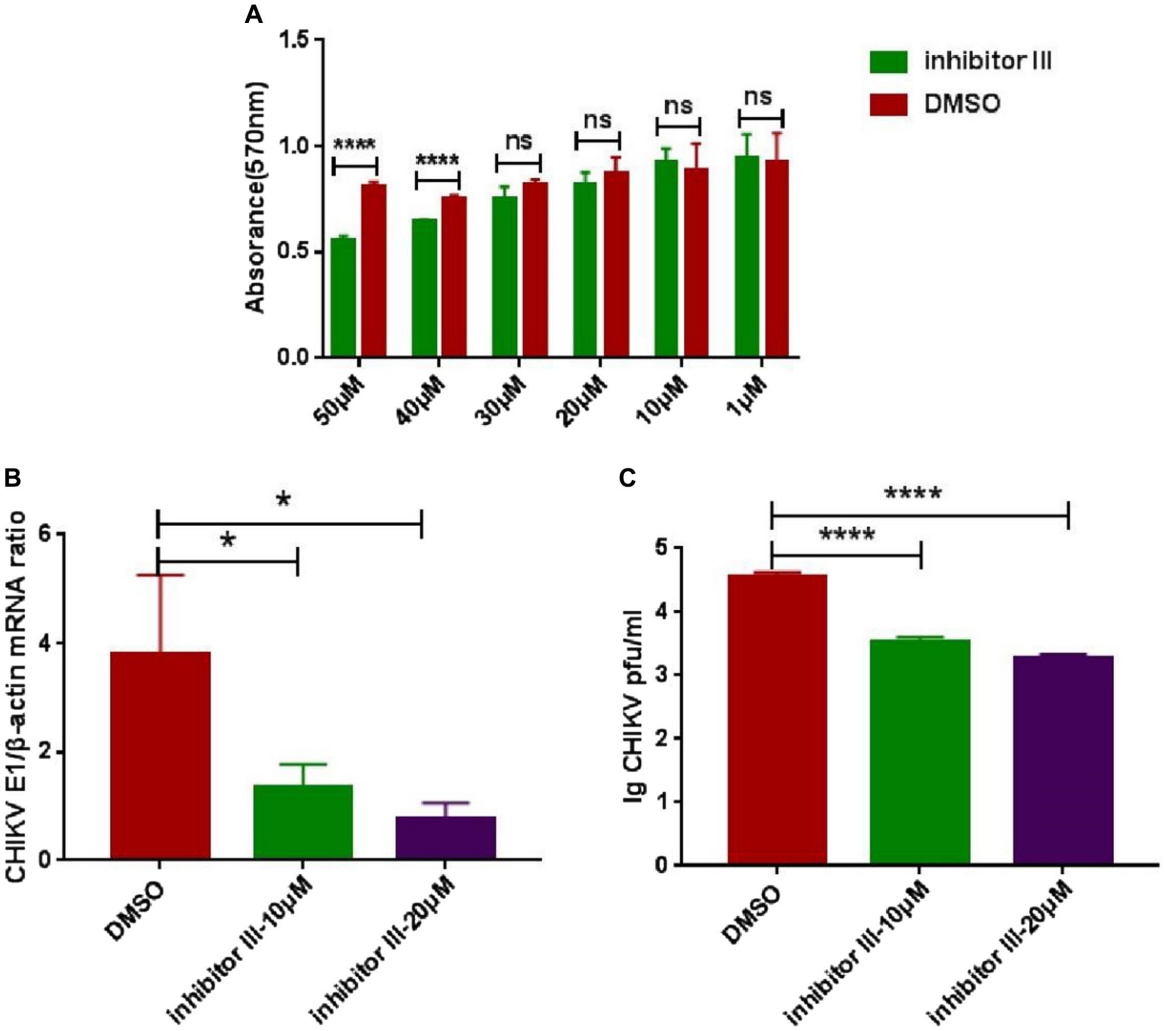
The cytotoxicity of inhibitor III and suppression effect of activity inhibitor III of calpain-2 protein on CHIKV replication. **(A)** The cytotoxicity of inhibitor III was detected by MTT assays at 48 h post-treatment. The absorbance value at 570 nm was measured after 48 h of treatment. **(B)** Hela cells were infected with CHIKV (0.1-MOI) and treated with 20 μM inhibitor III and 10 μM inhibitor III. The cells were collected at 48 h.p.i. The total RNA in the cells was extracted, and the expression level of CHIKV E1 gene mRNA was detected by qPCR amplification. **(C)** Hela cells were infected with CHIKV (0.1-MOI) and treated with 20 μM inhibitor III and 10 μM inhibitor III. The cell supernatant was collected at 48 h.p.i and analyzed by plaque assay to determine the virus titer. All data are presented as means ± SD from three independent experiments, each performed in triplicate. Statistical significance was expressed as the following: *p < 0.05; **p < 0.01; ***p < 0.001; ****p < 0.0001; and ns, not significant.

The cytotoxicity of inhibitor III was detected by MTT assays at 48 h post-treatment. The results showed that inhibitor III did not exhibit any cytotoxic effects in Hela cells when the concentration was no more than 30 μM (Figure 1A). When the concentration was 20 μM or 10 μM, inhibitor III could significantly reduce the expression level of CHIKV E1 gene mRNA in Hela cells and the viral titer in the supernatant (Figures 1B,C).

### The specific siRNA of calpain-2 protein could significantly inhibit the replication of CHIKV

**Figure 2:**
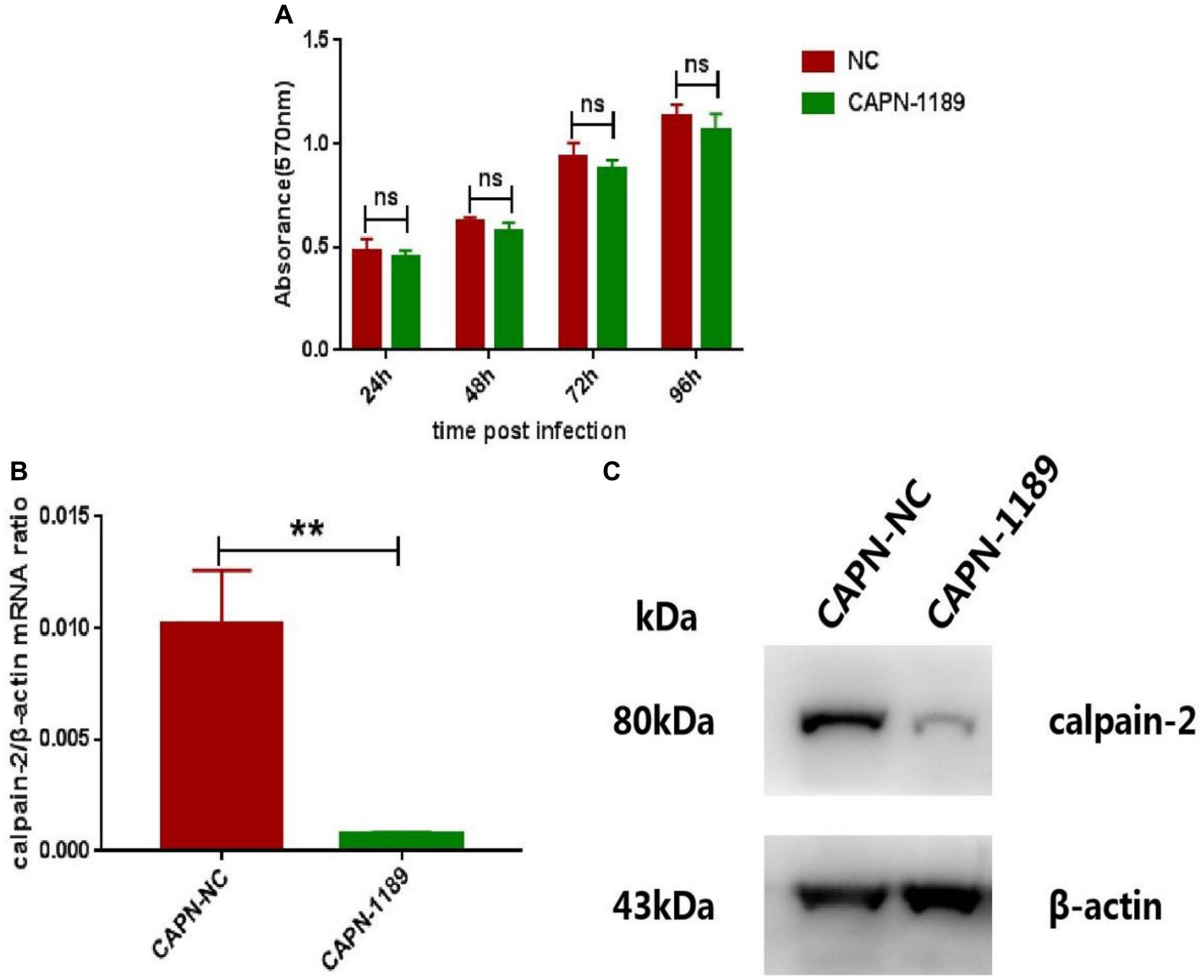
Verification of the cytotoxicity and knockdown effect of CAPN-1189. **(A)** The cytotoxicity of CAPN-1189 was detected by MTT assays. Hela cells were transfected with 40 nM of siRNAs. The mock transfection control was carried out with the addition of NC. The absorbance value at 570 nm was measured at 24, 48, 72 and 96 h post-transfection. **(B)** Hela cells were transfected with 40 nM CAPN-NC or CAPN-1189 against calpain-2 protein. Forty-eight hours after transfection, the total RNA in the cells was extracted, and the expression level of calpain-2 gene mRNA was detected by qPCR amplification. **(C)** Hela cells were transfected with 40 nM CAPN-NC or CAPN-1189 against calpain-2 protein. Forty-eight hours after transfection, cells were lysed and equal protein amounts were subjected to immunoblot analysis using a calpain-2 antibody (upper panel). β-actin was used as a loading control (lower panel). All data are presented as means ± SD from three independent experiments, each performed in triplicate. Statistical significance was expressed as the following: *p < 0.05; **p < 0.01; ***p < 0.001; ****p < 0.0001; and ns, not significant.

In order to verify whether reduced expression of calpain-2 protein would affect the replication of CHIKV, we used siRNA targeting calpain-2 protein named CAPN-1189 and nonsilencing siRNA called NC as a negative control. The potential cytotoxicity of siRNA was detected by MTT assays carried out at 24, 48, 72 and 96 h post-transfection. The results showed that CAPN-1189 did not display any cytotoxic effects in Hela cells (Figure 2A). Forty-eight hours post-transfection, the reduction of calpain-2 gene mRNA expression and protein levels was confirmed by qPCR and Western Blot analysis separately (Figures 2B,C).

**Figure 3:**
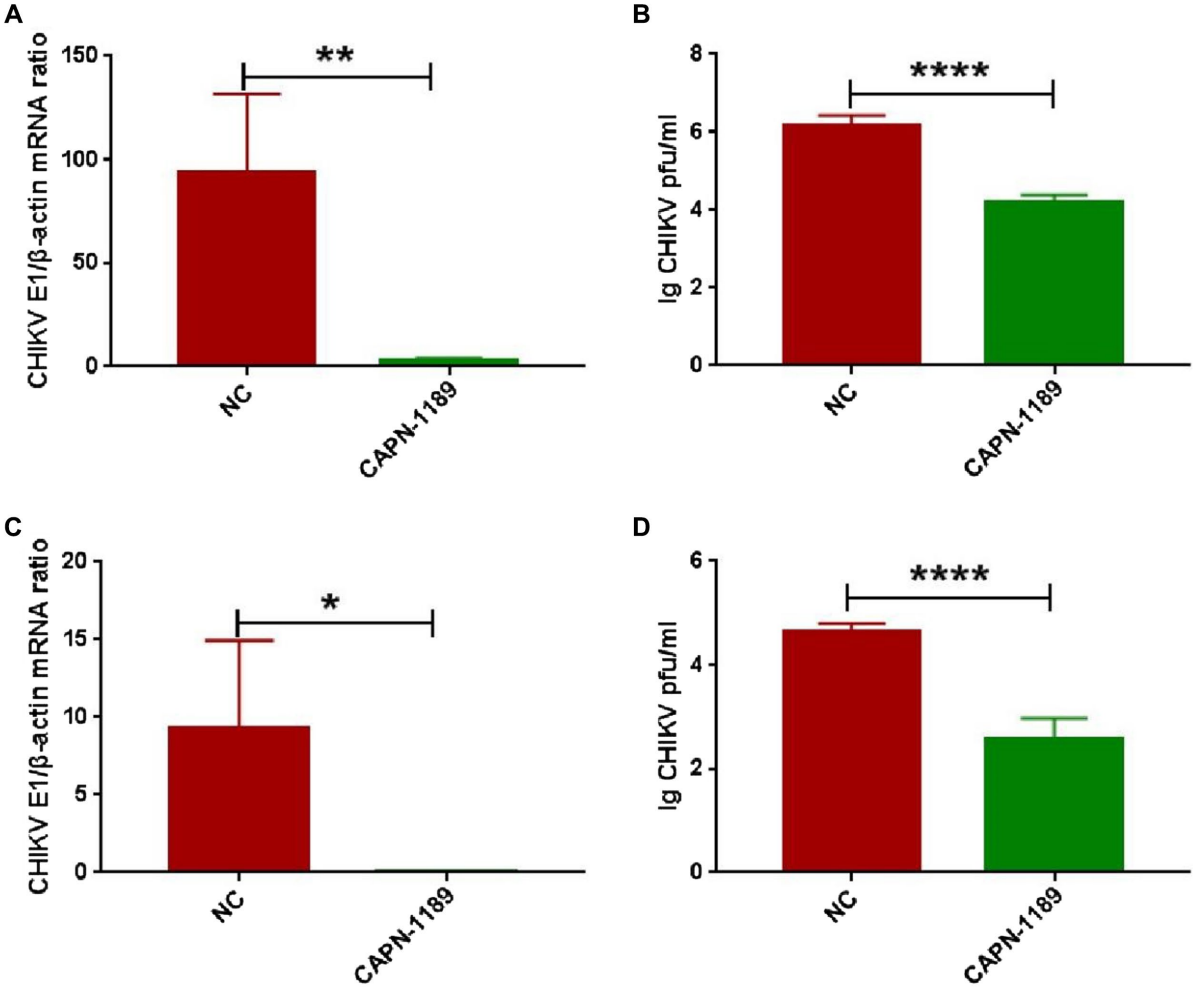
Effect of specific siRNA of calpain-2 protein on CHIKV replication. Hela cells were transfected with 40 nM CAPN-NC or CAPN-1189 against calpain-2 protein. Forty-eight hours later, Hela cells were infected with CHIKV (1-MOI) and then collected at 48 h.p.i. **(A)** The total RNA in the cells was extracted, and the expression level of CHIKV E1 gene mRNA was detected by qPCR amplification. **(B)** The cell supernatant was collected. The titer of CHIKV in Hela cell supernatant was detected by plaque assay. Hela cells were transfected with 40 nM CAPN-NC or CAPN-1189 against calpain-2 protein. Forty-eight hours later, Hela cells were infected with CHIKV (0.1-MOI) and then collected at 48 h.p.i. **(C)** The total RNA in the cells was extracted, and the expression level of CHIKV E1 gene mRNA was detected by qPCR amplification. **(D)** The cell supernatant was collected. The titer of CHIKV in Hela cell supernatant was detected by plaque assay. Statistical significance was expressed as the following: *p < 0.05; **p < 0.01; ***p < 0.001; ****p < 0.0001; and ns, not significant.

Forty-eight hours post-transfection with siRNA, Hela cells were infected with CHIKV. The expression level of CHIKV E1 gene mRNA was detected by qPCR amplification and the viral titers were determined by plaque assay. As shown in Figure 3, CHIKV E1 gene mRNA expression and titers were significantly reduced after siRNA knockdown of the calpain-2 protein.

### CHIKV infection did not cause significant changes in calpain-2 protein expression and activity

**Figure 4:**
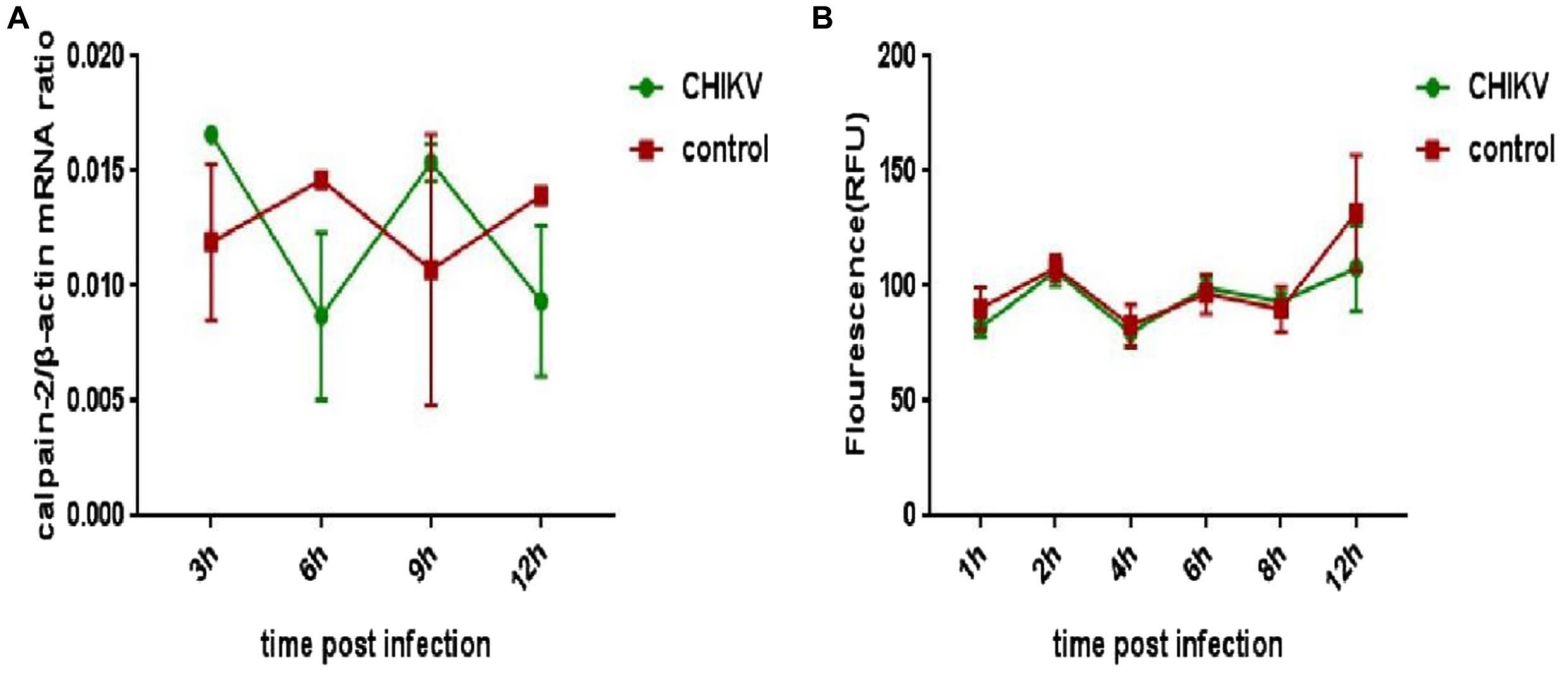
Changes of calpain-2 protein expression and activity after CHIKV infection. **(A)** Hela cells were infected with CHIKV (1-MOI). The cells were lysed at 48 h.p.i. The total RNA in the cells was extracted, and the expression level of calpain-2 gene mRNA was detected by qPCR amplification. **(B)** Hela cells were infected with CHIKV (1-MOI) and lysed at different time points. Equal cellular protein amounts were subjected to test calpain-2 protein activity. All data were presented as means ± SD from three independent experiments. Each was performed in triplicate.

To explore whether CHIKV infection would cause changes in the expression or activity of calpain-2 protein, Hela cells were inoculated with CHIKV. The cell lysate was collected at 48 h.p.i to extract nucleic acids for qPCR detection, and the cell protein extracted was tested for calpain-2 protein activity. As revealed in Figure 4, after CHIKV infected Hela cells, neither the expression nor the activity level of calpain-2 protein changed significantly.

### The activity inhibitor III of calpain-2 protein could inhibit the rearrangement of intracellular vimentin protein caused by CHIKV infection

Hela cells were treated with inhibitor III or DMSO, which were later infected by CHIKV, following by being fixed in confocal culture dishes at 6 h, 12 h and 24 h after the infection to be subjected to immunofluorescence experiments. The experiment was divided into three groups. Hela cells were neither infected by CHIKV nor treated with inhibitor III in “control” group; while the cells were infected by CHIKV and treated with DMSO in “DMSO” group. Finally, the cells were infected by CHIKV and treated with inhibitor III in “inhibitor III” group. The distribution of vimentin protein in Hela cells of different experimental groups was observed by confocal microscopy.

**Figure 5:**
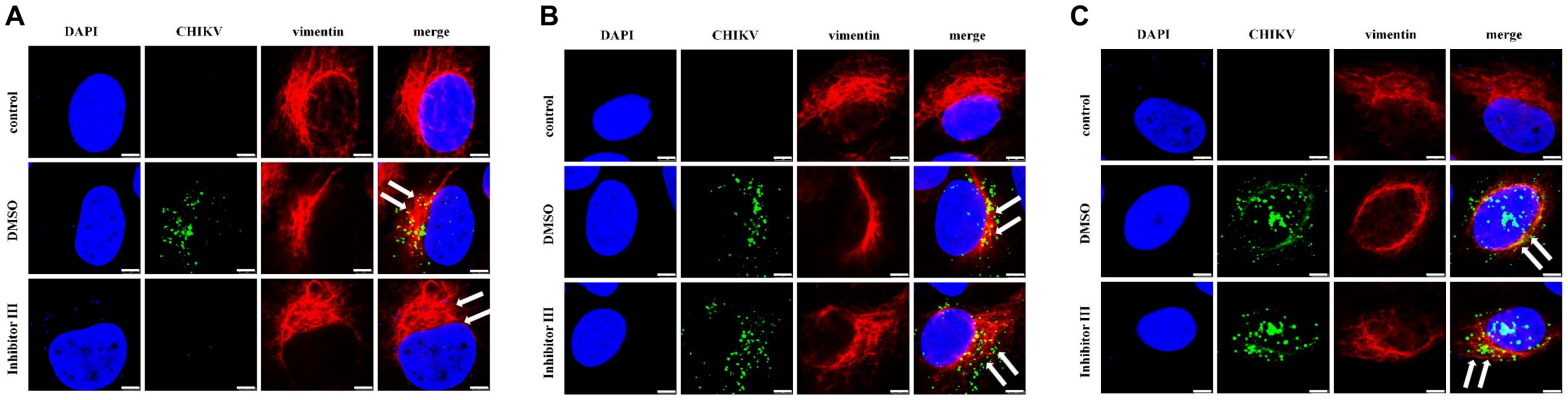
Distribution of vimentin protein in Hela cells at different time points after CHIKV infection. Hela cells were treated with 30 μM inhibitor III in advance and then infected by CHIKV (10-MOI) along with the treatment of inhibitor III. Hela cells were fixed at 6 h.p.i **(A)**, 12 h.p.i **(B)** and 24 h.p.i **(C)** for immunofluorescence detection. The distribution of vimentin in Hela cells was observed by laser confocal microscope. Among them, blue represented the nucleus stained by DAPI stain; green represented CHIKV; red represents vimentin; merge was an overlay of three different color channels (Scale bars, 5 μm).

The result showed that no detection of CHIKV was observed and the vimentin intermediate filaments remained well-spread to the cellular periphery in “control” group. Upon CHIKV infection, vimentin started to lose its usual filamentous structures and retracted from the plasma membrane, which became more obvious as the infection time increased in “DMSO” group. When inhibitor III was used along with CHIKV infection, the rearrangement of vimentin was suppressed in CHIKV-infected Hela cells in “inhibitor III” group. The vimentin became more well-spread to the cellular periphery than that in “DMSO” group (Figure 5).

**Figure 6:**
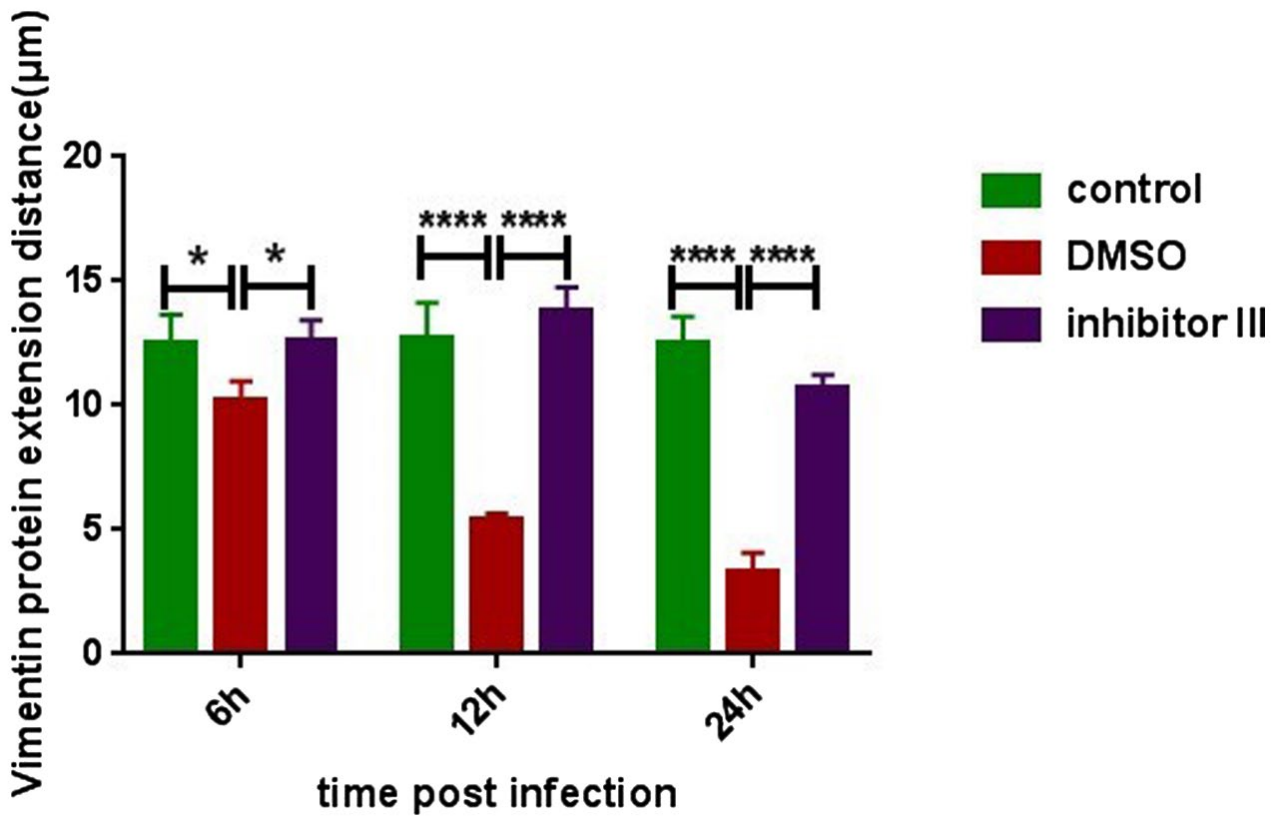
The distance from the outermost layer of vimentin protein to the edge of the nucleus at different time points after CHIKV infection. Hela cells were treated with 30 μM inhibitor III in advance and then infected by CHIKV (10-MOI) along with the treatment of inhibitor III. Hela cells were fixed at 6 h.p.i, 12 h.p.i and 24 h.p.i for immunofluorescence detection. The distance from the outermost layer of vimentin to the edge of the nucleus was detected. Statistical significance was expressed as the following: *p < 0.05; **p < 0.01; ***p < 0.001; ****p < 0.0001; and ns, not significant.

To further quantify the comparison, we randomly selected 6 Hela cells in each group and compared the distances between the outermost layer of vimentin protein in Hela cells and the edge of the nucleus in the above three groups (Figure 6). After CHIKV infection, the distance between the outermost layer of vimentin protein and the edge of the nucleus in Hela cells in “DMSO” group was significantly reduced compared with that in “control” group, as described previously, which became more obvious as the infection time increased. After the treatment of inhibitor III along with CHIKV infection, the distance in Hela cells in “inhibitor III” group was elongated compared with that in “DMSO” group.

**Figure 7:**
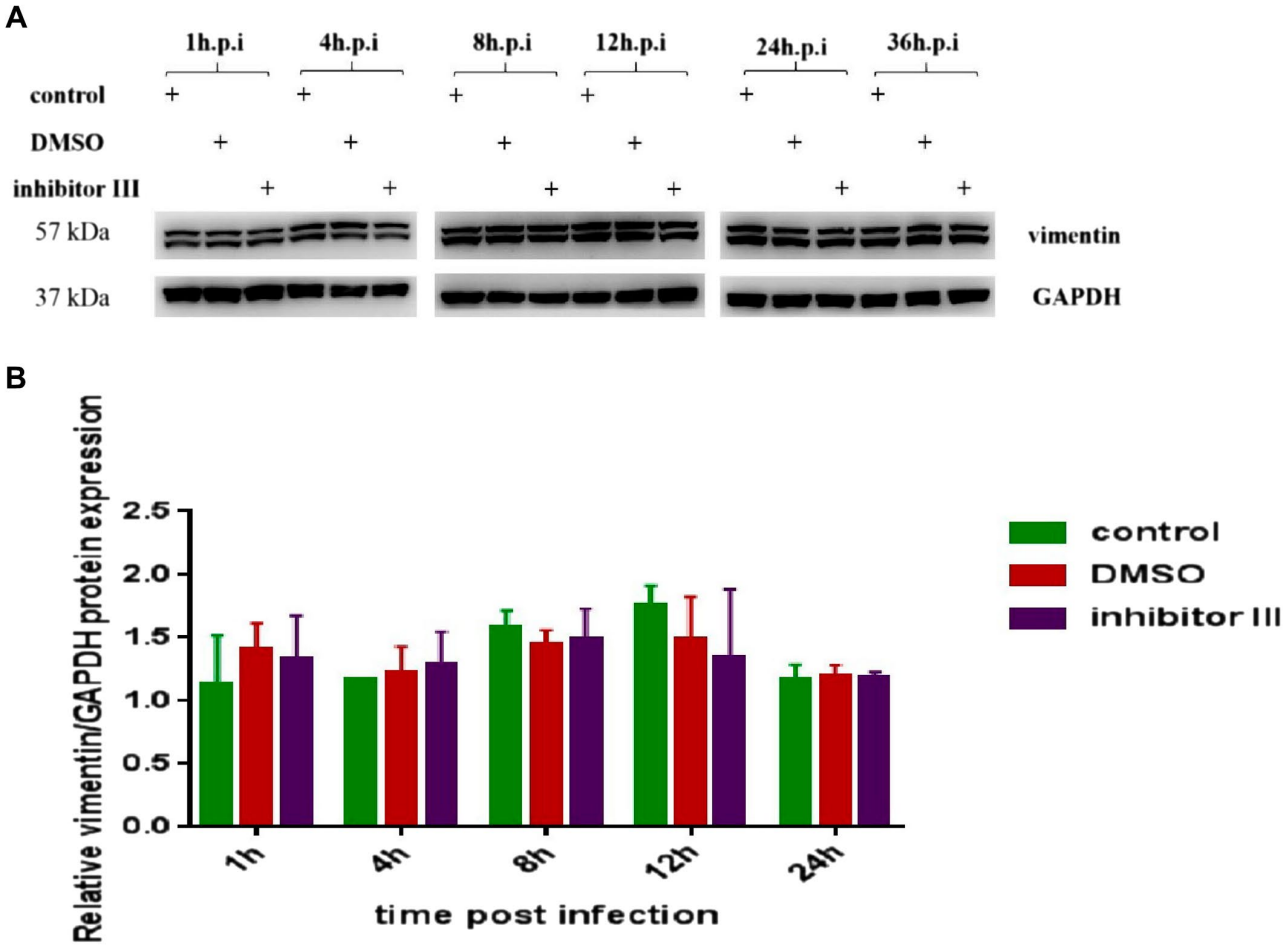
Expression of vimentin protein in Hela cells at different time points after CHIKV infection. **(A)** Hela cells were treated with 30 μM inhibitor III in advance and then infected by CHIKV (10-MOI) along with the treatment of inhibitor III. Equal cellular protein amounts were subjected to immunoblot analysis using a vimentin antibody (upper panel). GAPDH was used as a control (lower panel). **(B)** Expressions of vimentin protein were detected by Western blotting and quantified using ImageJ software. Statistical significance was expressed as the following: *p < 0.05; **p < 0.01; ***p < 0.001; ****p < 0.0001; and ns, not significant.

At the same time, we detected the expression level of vimentin protein in Hela cells in the above three groups by Western Blot. The results confirmed that neither CHIKV infection nor inhibitor III significantly changed the expression level of vimentin protein (Figure 7).

## Discussion

At present, there are no specific and effective drugs and vaccines for CHIF, and clinical treatment is mostly symptomatic. In the study, we had confirmed that calpain-2 protein was essential for the replication of CHIKV. And then we further explored the specific mechanism by which the calpain-2 protein affected the viral replication. The research results showed that CHIKV infection would cause the rearrangement of vimentin protein, which could be suppressed by inhibitor III, a kind of inhibitor for calpain-2 protein.

Calpain-2 protein have been reported previously to play a role in CHIKV replication (Karpe et al., 2016). Our data involving chemical inhibition of calpain-2 protein and specific siRNA knockdown strongly suggested that the suppression of either the activity or the express of calpain-2 protein would result in the inhibition of the replication of CHIKV. However, Cells infected with CHIKV did not show significant change in the express or activity of calpain-2 protein when compared to the noninfected control cells, which was accorded with the previous study. Researches have shown that the calpain-2 protein affects the replication process of a variety of viruses. Inhibiting the activity of calpain-2 protein or silencing the calpain-2 gene by calpain-2 protein-specific siRNA can significantly inhibit virus replication (Chung et al., 2005; Upla et al., 2008; Laajala et al., 2019). Intracellular replication of SARS-CoV can also be inhibited by calpain protein inhibitor MDL28170 and calpain-2 protein-specific siRNA (Schneider et al., 2012). In addition, specific siRNA gene silencing and overexpression of calpain inhibitor effectively suppressed IAV replication, IAV-triggered leukocyte infiltration, and the expression of pro-inflammatory mediators (including cytokines and chemokines) in mice, while greatly reduced mortality in animal models of IAV infection. Compared with calpain-1 protein, the effect of calpain-2 protein was more significant (Blanc et al., 2016). Besides, studies have shown that calpain protein is also associated with herpes simplex virus (HSV), hepatitis C virus (HCV) and some other small RNA viruses (Kalamvoki and Mavromara, 2004; Zheng et al., 2014; Howe et al., 2016). The replication of CHIKV, like other single-stranded positive-stranded RNA viruses such as EV1, SARS-CoV, needs the formation of replication complexes (RCs) in cell membranes or subcellular organelles, which depends on the activity of calpain-2 protein (Karpe et al., 2016).

Afterwards, we explored the specific mechanism by which the inhibitor III inhibited viral replication. Our results reflected that CHIKV infection led to vimentin remodeling and formation of cage-like structures, which became more pronounced as the infection time increased. When the inhibitor III came into play, the rearrangement of the vimentin protein was inhibited. Research has shown that the rearrangement and reorganization of vimentin intermediate filaments were induced by CHIKV-infection, which was consistent with our findings (Issac et al., 2014). However, our data showed that CHIKV infection did not cause changes in the vimentin protein expression levels, which might be due to cytoskeletal proteins acting more on their distribution rather than their expression levels.

Viral infections rewire cellular networks, especially endomembrane and cytoskeleton, to generate the viral RCs for efficient replication (Neufeldt et al., 2018). Rearrangement of vimentin protein was essential for viral assembly site formation by providing a mechanical scaffold to recruit viral proteins required for ASFV DNA replication (Stefanovic et al., 2005).

Studies also showed that vimentin reorganization was important for the maintenance and support of DENV RCs to facilitate efficient viral RNA replication. Because, gene silencing of vimentin by small interfering RNA induced significant alterations in RC distribution in DENV-infected cells (Teo and Chu, 2014). Additional studies found that vimentin filaments underwent dramatic reorganization during viral protein synthesis to form perinuclear cage-like structures containing and concentrating RCs using advanced imaging techniques, further revealing vimentin protein, as a structural element of RCs integrity, played an important role during the ZIKV infection (Zhang et al., 2022). Similarly, the interaction of vimentin protein with non-structural protein (nsP3) of CHIKV suggested the role played by vimentin protein during CHIKV replication by forming an anchorage network with the CHIKV replication complexes (RCs) (Issac et al., 2014).

Taken together, this explains our findings. As are well-known, calpain-2 protein plays an important role in the formation of viral replication complex, and vimentin is an indispensable component in the formation and stability of viral replication complex. Therefore, inhibiting the expression or activity of calpain-2 protein leading to a negative effect in CHIKV replication may accomplished though inhibiting the rearrangement of intracellular vimentin caused by virus-infected cells, which may provide new ideas and directions for antiviral strategies. The insufficiency is that we should use drugs or chemicals that inhibit the rearrangement of vimentin to corroborate our conjecture in subsequent studies, and we should further explore the way or pathway by which calpain-2 protein affects the rearrangement of vimentin.

## Conclusion

So far, CHIKV is spreading globally, while its specific pathogenic mechanism is still a scientific issue worth exploring, which is important for understanding the biological significance and the future development of antiviral strategies. In this study, we confirmed that inhibiting the expression or activity of calpain-2 protein may suppress CHIKV replication and repress the intracellular vimentin rearrangement caused by CHIKV infection, and this research results may provide new directions and targets for future antiviral strategies.

## Data availability statement

The original contributions presented in the study are included in the article/supplementary material, further inquiries can be directed to the corresponding authors.

## Ethics statement

The animal study was approved by Ethics Committee of Southern Medical University (K2021016). The study was conducted in accordance with the local legislation and institutional requirements.

## Author contributions

ZR designed the experiments. JL drafted the manuscript. KZ and HS performed experiments and analyzed cell experimental data. JL, KZ, and HW analyzed experimental results and data. ZL and CW guided the design of the study and revised the manuscript. All authors read and approved the final manuscript, contributed to the article and approved the submitted version.
